# Coexistence of Fosfomycin and Colistin Resistance in *Klebsiella pneumoniae*: Whole-Genome Shotgun Sequencing

**DOI:** 10.1128/genomeA.01303-16

**Published:** 2016-11-23

**Authors:** Balaji Veeraraghavan, Susmitha K. Perumalla, Naveen Kumar Devanga Ragupathi, Agila Kumari Pragasam, Dhiviya Prabaa Muthuirulandi Sethuvel, Sandhana Inian, Francis Yesurajan Inbanathan

**Affiliations:** Department of Clinical Microbiology, Christian Medical College, Vellore, India

## Abstract

Resistance to colistin is a major threat that limits therapeutic choices for treating carbapenem-resistant *Klebsiella pneumoniae* infections. Herein, we report the draft genome sequences of two colistin-resistant *K. pneumoniae* isolates (BA41763 and B6753). The sequence data indicate that BA41763 and B6753 contain genomes of ~5.9 and 5.7 Mb in size with several plasmids.

## GENOME ANNOUNCEMENT

Carbapenem-resistant *Klebsiella pneumoniae* (CRKP) infections are difficult to manage, with very few remaining treatment options, such as colistin and tigecycline (1). Due to inappropriate/enormous use, emergence of colistin resistance has become a serious concern to the microbiologists and clinicians (2), especially in developing countries like India. Hence, there is a great need for the screening of colistin resistance in Indian settings. In this study, we determined the draft genome sequences of CRKP isolates (BA41763 and B6753) from bloodstream infections. Both isolates were resistant to ceftazidime, cefotaxime, cefoxitin, cefepime, piperacillin-tazobactam, cefoperazone/sulbactam, imipenem, meropenem, ciprofloxacin, gentamicin, netilmicin, amikacin, and tigecycline except polymyxin B 300. Interestingly, the isolates were resistant to colistin, with MICs of 1024 and 16 µg/ml, respectively, for BA41763 and B6753 by the broth microdilution method.

To further understand the molecular mechanism behind colistin resistance, we determined their whole-genome shotgun sequences in Ion Torrent PGM using 400-bp chemistry. *De novo* assembly of data was performed using AssemblerSPAdes v5.0.0.0 in Torrent suite server v5.0.3. The assembled genomes of BA41763 and B6753 resulted in 147 and 116 contigs (≥500 bp) with 49× and 23× coverage, respectively. Assembled genome sequences were annotated in PATRIC, the bacterial bioinformatics database and analysis resource (http://www.patricbrc.org) (3), Rapid Annotations using Subsystems Technology (RAST) pipeline (http://rast.nmpdr.org/) (4, 5), and NCBI Prokaryotic Genomes Automatic Annotation Pipeline (PGAAP, http://www.ncbi.nlm.nih.gov/genomes/static/Pipeline.html).

The genome of BA41763 had 5,944,266 bp with 6,678 coding sequences (CDS), 10 rRNAs, and 66 tRNAs. The annotation revealed 32 and 85 ARDB and CARD antimicrobial resistance genes, respectively. In addition, 74 and 183 virulence factors from VFDB and the Victors database, respectively, were identified (https://www.patricbrc.org). Similarly for B6753, 5,754,328 bp with 6,248 CDS were identified. Also identified were 13 rRNAs, 72 tRNAs with 34 and 82 ARDB and CARD antimicrobial resistance genes and 72 and 181 virulence factors from the VFDB and Victors database, respectively.

Next-generation sequencing (NGS) also revealed that BA41763 and B6753 were of sequence types (STs) ST147 and ST14, respectively, as analyzed by the MLST 1.8 tool (https://cge.cbs.dtu.dk//services/MLST/) (6). In BA41763, ResFinder 2.1 from the CGE server (https://cge.cbs.dtu.dk//services/ResFinder/) (7) revealed the presence of aminoglycoside (*rmt*f and *aac*A4), beta-lactam (*bla*TEM-1B, *bla*CTX-M-15), fluoroquinolones (*oqx*B, *aac*(6′)*lb-cr*, *oqx*A, *qnr*B66), fosfomycin (*fos*A), macrolide (*mph*(A)), rifampin (ARR-2), and sulfonamide (*sul*2) resistance genes. Similarly, for B6753, we found aminoglycoside (*aad*A2, *aac*A4, *arm*A, *aph*(3′)-Vla), beta-lactam (*bla*OXA-1, *bla*CTX-M-15, *bla*OXA-232, *bla*NDM-1, *bla*SHV-28, *bla*TEM-1A), fluoroquinolone (*aac*(6′)*lb-cr*), fosfomycin (*fos*A), macrolide (*mph*(E), *msr*(E)), phenicol (*cat*B3), sulfonamide (*sul*1), tetracycline (*tet*(D), and trimethoprim (*dfr*A14, *dfr*A12). However, plasmid-mediated colistin resistance genes mcr-1 and mcr-2 were absent. B6753 had a mutation in the *mgrB* gene with a premature stop codon resulting in a truncated protein of 27 amino acids, but there were no mutations in the *mgr*B gene of BA41763.

PlasmidFinder 1.3 (https://cge.cbs.dtu.dk//services/PlasmidFinder/) (8) revealed the presence of ColKP3, IncFIB (Mar), and IncHI1B plasmids common in BA41763 and B6753. In addition, BA41763 was found with IncFIB (pKPHS1), IncHI1B, IncFII (pKPX1), ColpVC, and IncR plasmids, whereas B6753 was positive for the IncFIB(K) plasmid. Overall, the mechanism of colistin resistance seemed to be chromosomally mediated. However, screening of plasmid-mediated colistin resistance (mcr-1 and mcr-2) is of utmost significance among CRKP organisms, to prevent further spread of colistin resistance.

### Accession number(s).

This whole-genome shotgun project of both the isolates BA41763 and B6753 has been deposited at DDBJ/ENA/GenBank under the accession numbers LZYN00000000 and MEBR00000000, respectively. The versions described in this paper are the first versions, LZYN01000000 and MEBR01000000.
